# Differential transforming activity of the retroviral Tax oncoproteins in human T lymphocytes

**DOI:** 10.3389/fmicb.2013.00287

**Published:** 2013-09-23

**Authors:** Tong Ren, Hua Cheng

**Affiliations:** ^1^Penn State Hershey Cancer InstituteHershey, PA, USA; ^2^Institute of Human Virology, School of Medicine, University of MarylandBaltimore, MD, USA

**Keywords:** HTLV-1/-2, Tax, human T lymphocytes, immortalization, transformation

## Abstract

Human T cell leukemia virus type 1 and type 2 (HTLV-1 and -2) are two closely related retroviruses. HTLV-1 causes adult T cell leukemia and lymphoma, whereas HTLV-2 infection is not etiologically linked to human disease. The viral genomes of HTLV-1 and -2 encode highly homologous transforming proteins, Tax-1 and Tax-2, respectively. Tax-1 is thought to play a central role in transforming CD4+ T lymphocytes. Expression of Tax-1 is crucial for promoting survival and proliferation of virally infected human T lymphocytes and is necessary for initiating HTLV-1-mediated oncogenesis. In transgenic mice and humanized mouse model, Tax-1 has proven to be leukemogenic. Although Tax-1 is able to efficiently transform rodent fibroblasts and to induce lymphoma in mouse model, it rarely transforms primary human CD4+ T lymphocytes. In contrast, Tax-2 efficiently immortalizes human CD4+ T cells though it exhibits a lower transforming activity in rodent cells as compared to Tax-1. We here discuss our recent observation and views on the differential transforming activity of Tax-1 and Tax-2 in human T cells.

HTLV-1 and -2 are two highly homologous human retroviruses. Both viruses are blood-borne pathogens, which can be transmitted through perinatal transmission by blood contamination or breast milk feeding, sexual transmission, exposure to contaminated blood products or sharing contaminated needles (Clark et al., 1985; The T- and B-cell Malignancy Study Group, 1988). Although both HTLV-1 and -2 utilize GLUT-1, a ubiquitously expressed cell surface (SU) protein, as the receptor for viral entry (Igakura et al., 2003; Manel et al., 2003; Coskun and Sutton, 2005), they exhibit distinct cell tropism. HTLV-1 preferentially infects and transforms CD4+ T cells, whereas HTLV-2 has tropism for CD8+ T cells, which suggests that there exists a CD8+ T cell-specific co-factor for assisting viral entry and permissive replication of HTLV-2 (Ijichi et al., 1992). HTLV-1 is the etiological factor that causes adult T cell leukemia and lymphoma (Hattori et al., 1981; Hinuma et al., 1981, 1982; Yamaguchi et al., 1984). HTLV-2 was detected in CD8 T cells from a patient with hairy cell leukemia, a rare type of leukemia that affects B lymphocytes (Kalyanaraman et al., 1982), however the causative link between HTLV-2 infection and hairy cell leukemia has not been established. Thus far, HTLV-2 infection has not been etiologically linked to any human disease.

HTLV-1 viral genome is known to encode two oncogenic products, Tax-1 and HBZ (Satou et al., 2006; Satou and Matsuoka, 2012). Tax-1 is the first recognized viral component to display oncogenic potential. Evidence showed that Tax-1 plays an essential role in mediating transformation of T lymphocytes (Tanaka et al., 1990). The transforming activity of Tax-1 in lymphocytes was first demonstrated using HTLV-1 infectious molecular clone. The wild type molecular clone efficiently transformed human primary T cells, while the molecular clone with disrupted *tax* gene failed to do so, defining the role of Tax-1 as a viral transforming protein (Yamaoka et al., 1996, 1998; Akagi et al., 1997; Matsumoto et al., 1997; Iwanaga et al., 1999). However, this experiment did not rule out potential participation of other viral components in HTLV-1-mediated cell transformation. Unexpectedly, when expressed alone, Tax-1 rarely immortalizes or transforms terminally differentiated T cells derived from human peripheral blood (Bellon et al., 2010). In humanized mouse model, Tax-1-expressing human CD34+ blood progenitor cells, when transplanted into NOD/SCID mice, developed T cell lymphoma of human origin (Banerjee et al., 2010). This experimental finding suggests that in some cases, leukemic cells might be evolved from HTLV-1-infected CD34+ blood progenitor cells rather than from infected mature T cells. This assumption is yet to be validated.

A comparative study on two highly homologous Tax proteins from HTLV-1 and -2 is crucial in deciphering the molecular pathogenesis of HTLV-1-associated ATL. We expressed the Tax proteins in human T cells isolated from peripheral blood to imitate the process of HTLV-1 infection. This prospective approach would allow us to evaluate sequential oncogenic events in Tax-mediated proliferation, immortalization and transformation of human primary T cells. Normal lymphocytes isolated from healthy donors are typically at quiescent stage. Stimulation with mitogens such as PHA, recombinant IL-2 or mitogenic anti-CD3/-CD28 antibodies results in rapid proliferation of peripheral blood lymphocytes with displaying lymphoblastic morphology. Activated lymphocytes stop growing 2–3 weeks following stimulation at normal culture conditions by reaching the checkpoint of T cell senescence. HTLV-1 infection overrides replicative senescence of T cells, promotes continuous growth of HTLV-1-infected T cells and induces immortalization, ultimately resulting in IL-2-independent growth of the transformed T cells. At each step of aberrant proliferation, immortalization or transformation during HTLV-1 infection of T cells, a variety of cellular oncogenic alterations are expected to occur. Thus, studying these processes will provide crucial insights into the pathogenesis of ATL.

We previously hypothesized that in the absence of other viral proteins, Tax-1 is sufficient to immortalize human mature CD4+ T cells while Tax-2 preferentially immortalizes CD8+ T cells. We unexpectedly found that both Tax-1 and Tax-2 failed to immortalize human primary CD8+ T cells, and Tax-2 was able to immortalize human CD4+ T cells more efficiently than Tax-1 in the study with including 12 healthy blood donors. A similar finding was reported later (Imai et al., 2013). In addition, a majority of Tax-2-immortalized CD4+ T cell lines grew *in vitro* at the growth rate similar to some lymphoblastic leukemia cells (Ren et al., 2012). In contrast, Tax-1-immortalized CD4+ T cells were slow growing and exhibited spontaneous cell death at normal culture conditions (Ren et al., unpublished data). These experimental results were unlikely caused by technical variation because Tax-1 and Tax-2, in which their expressions were driven from human elongation factor promoter, were introduced into human primary T cells via VSV-G pseudotyped lentiviruses at similar efficiency.

Tax-2 appears to be more oncogenic than Tax-1 in primary CD4+ T cells when they are expressed alone, however Tax-1 is clearly oncogenic in CD4+ T cells in the context of an intact proviral clone and other expressed viral proteins. Although both Tax proteins are highly homologous, Tax-1 does exhibit distinct structural features in which Tax-2 lacks. Tax-1 contains a PDZ binding motif (PBM) in its carboxyl-terminus that is important for binding to DLG-1 and other PDZ containing proteins, and these interactions were thought to play an important role for cell transformation by Tax-1. In addition, Tax-1, but not Tax-2, undergoes K63-linked polyubiquitination as part of its mechanism to activate NF-κB (Shembade et al., 2007; Journo et al., 2013). It is apparent that these differences do not account for the stronger transforming capability of Tax-2 in primary CD4+ T cells. Tax-1 may acquire a full transforming ability in human CD4+ T cells in cooperation with HBZ, an HTLV-1 antisense gene product that is constitutively transcribed and remains intact in ATL cells. In fact, HBZ itself was proven leukemogenic in mouse model. 30% of HBZ-transgenic (Tg) mice developed T cell lymphoma and most of HBZ Tg mice developed spontaneous inflammatory lesions (Satou and Matsuoka, 2012). Furthermore, increasing evidences showed that the functional interaction between Tax and HBZ is crucial for HTLV-1 oncogenesis in patients. Since high levels of Tax-1 expression lead to hyper-activation of NF-κB, consequently resulting in replicative senescence or even cell death (Zhi et al., 2011), HBZ antagonizes Tax-1’s toxic function to facilitate immune evasion and to promote cell cycle progression and proliferation. Interestingly, Tax-2 also induces hyper-activation of NF-κB in CD4+ T cells, and it is capable of promoting T cell proliferation in the absence of the antisense gene product encoded from HTLV-2. This suggests that unlike Tax-1, Tax-2 is a singly important viral component of the HTLV-2 genome to execute its transforming activity in CD4+ T cells.

We also found that the Tax proteins had their own preference on donor selection, and Tax-1 and Tax-2 immortalized CD4+ T cells from different blood donors. In one unusual case, Tax-1 immortalized CD4+ T cells while Tax-2 immortalized CD4+ myeloid dendritic cells (mDCs) from CD4+ cell pools of the same blood donor (Ren et al., unpublished data). These mDCs showed negative lineage markers (CD3-/TCRαβ-/TCRγδ-/CD19-/CD56-/CD16-/CD14-/CD34-) and positive myeloid/dendritic cell markers (CD11c+/CD123+/HLA-DR+/CD2+/CD71+/CD117+/CD33+). Because the number of mDCs in healthy donors is typically less than 1%, the finding that Tax-2 is able to immortalize mDCs from enriched CD4+ pools suggests that the Tax-2-mediated immortalization of blood cells is highly selective. Aside from this finding, genotyping analysis of one Tax-2-established CD4 T cell line showed that these cells were clonal population. Together, our data demonstrate that in the absence of other viral components, Tax-2 alone is sufficient to promote clonal expansion of selected subsets of human primary CD4+ T cells. The potential cellular factor that contributes to Tax’s selectivity is currently unclear, which will certainly be an interesting subject for further investigation.

It is known that HTLV-1 has preferential tropism for CD4 T cells in healthy carriers, HAM/TSP and ATL patients. In addition to utilization of GLUT1 as an entry factor for HTLV viruses, both viruses also utilize NRP1. HTLV-1 and -2 have a differential requirement for heparan sulfate proteoglycans (HSPGs). Distinct from HTLV-1, HTLV-2 is not dependent on HSPGs for cell entry (Jones et al., 2006). Because HTLV-1 utilizes a ubiquitously expressed receptor for viral entry, it is expected that this virus would have the capacity to infect cell types other than CD4+ T cells. Indeed, CD8+ lymphocytes, monocytes and B-lymphocytes are found to harbor HTLV-1 (Koyanagi et al., 1993; Eiraku et al., 1998; Nagai et al., 2001). In addition, macrophages, dendritic cells, megakaryocytes as well as glial cells (astrocytes and microglial cells) are also the cell targets for HTLV-1 infection *in vivo *(Macatonia et al., 1992; Koyanagi et al., 1993; Levin et al., 1997; Grant et al., 2002). HTLV-1 isolates can be transmitted to primary human endothelial cells and basal mammary epithelial cells *in vitro *(Ho et al., 1984; Hoxie et al., 1984). Regardless of a broad cell tropism of HTLV-1, this virus exclusively causes malignant transformation of infected CD4+ T cells, suggesting that a panel of CD4 cell-specific cellular factors is crucial in assisting HTLV-1-mediated oncogenesis. Conversely, it is also possible that other types of cells may express cellular repressors to restrict HTLV-1 oncogenesis as seen in other retroviruses. For instance, SAMHD1 is able to restrict HTLV-1 replication in myeloid cells (St. Gelais and Wu, 2011). Unlike HTLV-1, HTLV-2 preferentially infects CD8+ T cells in human. However, Tax-2, when expressed by a pseudotype form of lentivirus, executes its transforming activity in CD4+ T cells. This phenomenon of disintegrated cell tropism (in CD8+ T cells) and disease-causing capacity (in CD4+ T cells) may partially explain why HTLV-2 does not currently cause leukemia in human.

These findings may have important implications on pathogenic virus evolution. Many viruses are considered non-pathogenic, because they do not apparently cause human disease based on current data. These non-pathogenic viruses usually do not receive much attention as known pathogenic viruses do. From an evolutionary point of view, recombination and cell tropism switch among homologous pathogenic and non-pathogenic viruses may occur, potentially generating a more lethal virus. A recent study demonstrated that cell tropism switch could be initiated by artificially generating chimeric envelope proteins. Switch of the envelope SU domains between HTLV-1 and HTLV-2 alters the tropism of HTLV-1 from CD4 to CD8+ T cells (Kannian et al., 2013). In the case of HTLV-2, the infection rate among IV drug abusers is as high as 30%. Since no treatment is available for HTLV-2 infection and host immunity is not sufficient to eradicate this virus, it is possible that the rate of infection is going to rise due to the presence of accumulative HTLV-2 reservoirs in host CD8+ T cells. Indeed, co-infection of HTLV-2 with HTLV-1 or with HIV-1 has been documented and is increasing (Uchiyama et al., 1977; Shimoyama, 1991), probably because these viruses share similar transmission pathways and risk factors. HTLV-2 could be eventually modified in dendritic cells or monocytes that are co-infected with HTLV-1 or HIV-1 to acquire new cell tropism for CD4+ T cells, leading to its transformation of CD4+ T cells. Although naturally occurring cell tropism switch among homologous viruses may take unpredictable amount of time, this event does occur evolutionarily.

Although Tax-2 does not transform normal CD8 T cells, this viral protein exhibits its transforming activity in CD8+ T cells derived from T cell type of large granular lymphocyte leukemia (T-LGLL). T-LGL leukemia is the malignancy of CD8+ cytotoxic T cells, which usually occurs in elderly patients. The disease course is frequently associated with autoimmune diseases (Rose and Berliner, 2004). Primary T-LGL leukemia cells display the CD3+/CD8+/CD57+ SU markers, representing activated cytotoxic T lymphocytes. Despite T-LGLL cells are leukemic cells, these cells do not grow in culture. By expressing Tax-2 in these cells, long-term growth and clonal expansion of T-LGLL cells could be achieved (Ren et al., unpublished data).

In summary (see **Table 1**), Tax-1 and Tax-2 display differential transforming activities in human T lymphocytes. Tax-2 demonstrates a more potent activity than Tax-1 in immortalizing human terminally differentiated CD4+ T cells. Besides, Tax-2, not Tax-1, is able to transform CD8+ T cells from T-LGL leukemic cells with preexisting oncogenic profile. The ability of Tax-2 to establish specific subsets of T cell lines implicates its utilization in studying human T cell biology and in developing leukemia model. Furthermore, the hypothesis that Tax-2 has a disease-causing capacity in CD4+ T cells needs to be validated in humanized mouse model.

**Table 1:** Differential activities of Tax-1 and Tax-2 in human T cells.

	Tax-1	Tax-2
**Activation of**
IKK/NF-kB	+	+
Stat3	+	+
PI3K/Akt	+	+
AP-1	+	+
CREB	+	+
Dysregulation of autophagy	+	+
Lipid raft involvement	+^a^	-
**Immortalization of**
CD4+ T cells	+^b^	+++^c^
CD8+ T cells	-	-
CD8+ T cells from T-LGLL	-	_+_

a Tax-1 recruits I κB kinases into lipid raft microdomains for persistent activation of NF-κB signaling, while Tax-2 activation of NF-κB does not appear to involve lipid rafts (Huang et al., 2009).

b Tax-1 is able to immortalize human primary CD4+ T cells with low efficiency (2 out of 12), and Tax-1-immortalized T cells grow slowly in culture and experience spontaneous cell death.

c Tax-2 immortalizes human primary CD4+ T cells more efficiently than Tax-1 (4 out of 12). Tax-2-immortalized T cells grow in culture at a rate comparable to some lymphoblastic leukemia cells with healthy growth appearance.

## Conflict of Interest Statement

The authors declare that the research was conducted in the absence of any commercial or financial relationships that could be construed as a potential conflict of interest.
